# Inhibition of Advanced Glycation End-Product Formation and Antioxidant Activity by Extracts and Polyphenols from *Scutellaria alpina* L. and *S. altissima* L.

**DOI:** 10.3390/molecules21060739

**Published:** 2016-06-14

**Authors:** Izabela Grzegorczyk-Karolak, Krzysztof Gołąb, Jakub Gburek, Halina Wysokińska, Adam Matkowski

**Affiliations:** 1 Department of Biology and Pharmaceutical Botany, Medical University of Lodz, ul. Muszynskiego 1, Lodz 90–151, Poland; halina.wysokinska@umed.lodz.pl; 2 Department of Pharmaceutical Biochemistry, Wroclaw Medical University, ul. Borowska 211A, Wroclaw 50-556, Poland; krzysztof.golab@umed.wroc.pl (K.G.); jakub.gburek@umed.wroc.pl (J.G.); 3 Department of Biology and Pharmaceutical Botany, Wroclaw Medical University, ul. Borowska 211, Wroclaw 50-556, Poland; bbsekret@umed.wroc.pl

**Keywords:** advanced glycation end products, flavonoids, antioxidants, *Scutellaria alpina*, *Scutellaria altissima*

## Abstract

Methanolic extracts from the aerial parts and roots of two *Scutellaria* species, *S. alpina* and *S. altissima*, and five polyphenols from these plants demonstrated a significant ability to inhibit the formation of advanced glycation end-products (AGE) *in vitro*. *S. alpina*, which is richer in polyphenolic compounds, had strong antiglycation properties. These extracts demonstrated also high activity in the FRAP (ferric-reducing antioxidant power), antiradical (DPPH) and lipid peroxidation inhibition assays. Among the pure compounds, baicalin was the strongest glycation inhibitor (90.4% inhibition at 100 μg/mL), followed by luteolin (85.4%). Two other flavone glycosides had about half of this activity. Verbascoside was similar to the reference drug aminoguanidine (71.2% and 75.9%, respectively). The strong correlation observed between AGE inhibition and total flavonoid content indicated that flavonoids contribute significantly to antiglycation properties. A positive correlation was also observed between antiglycative and antioxidant activities. The studied skullcap species can be considered as a potential source of therapeutic agents for hyperglycemia-related disorders.

## 1. Introduction

Glycation is the non-enzymatic formation of adducts between amino groups and the carbonyl groups of reducing sugars. In the early stage, a sugar reacts with the free amino group of a biological amine to form an unstable compound, a Schiff base, which then undergoes rearrangement to form more stable Amadori products (early glycation products). These Amadori products degrade to form a variety of reactive dicarbonyl compounds, such as glyoxal, methyl glyoxal and deoxyglucosones, which are much more reactive than the initial sugars. In the late stage of glycation, due to dehydration, oxidation and cyclization reactions, irreversible compounds, called advanced glycation end products (AGEs), are formed [1].

The accumulation of protein glycation products in living organisms leads to structural and functional modifications of tissue proteins. Via receptor-dependent and direct pathways, AGEs promote the initiation and development of diabetic complications, which are categorized as macro- and micro-vascular lesions. The most common microvascular complications include neuropathy, nephropathy, retinopathy and cataract, while the macrovascular complications are atherosclerosis, coronary heart disease and cerebrovascular disease [2,3,4]. Although hyperglycemia plays a key role in the pathogenesis of diabetic complications, the formation of AGE progressively increases with normal aging.

Traditionally, plant extracts have been used in the management of diabetes. Numerous studies have shown the effectiveness of crude plant extracts or their bioactive compounds in lowering blood glucose levels [5]. The inhibition of advanced glycation end products (AGEs) is yet another mode of diabetes treatment not dependent on the control of blood glucose level and would be useful in the prevention or mitigation of certain diabetic complications [6,7]. AGE inhibitors are divided into three classes: carbonyl-trapping agents, which attenuate carbonyl stress; metal ion chelators, which suppress glycoxidation; and cross-link breakers, which reverse AGE cross-linking [8]. Many synthetic inhibitors of AGE formation are not in clinical use, due to unsatisfactory safety profiles. However, the phenolic phytochemicals present in fruits, herbs and vegetables, besides being strong antioxidants and glycation inhibitors, are usually safe for human consumption [9,10].

Several species of skullcap (genus *Scutellaria*) have been used in folk medicine for thousands of years. They have been traditionally used as anti-inflammatory, antiviral, sedative, antithrombotic and antioxidant remedies, primarily in East Asia. Skullcaps were prepared as a tincture alone and with other herbs in numerous prescriptions, such as: Huangqui Baizhi San, Huangqui Yin, Huangqui Tang, Huangqui Gao. The tea industry used *S. baicalensis* also for making tea and in the food industry as a flavoring agent for stewing meat and fish [11]. Some species and the phenolic compounds isolated from them continue to be used also in conventional medicine [11]. One species, *S. baicalensis*, is listed in European and Chinese pharmacopoeias.

The two species chosen for our study, *S. altissima* L. and *S. alpina* L., are native to some regions in Eastern and Southern Europe and are not fully exploited as official medicinal herbs. However, they are known to contain substantial amounts of potentially highly active polyphenols. At present, the only information on antiglycation activity is available for the well-known traditional Chinese herb *Scutellariae baicalensis radix* [12]. The two major flavones present in *S. baicalensis*, baicalin and wogonoside, are also present in both studied species [13]. The present paper reports the *in vitro* antiglycative and antioxidant activity of extracts from the roots and aerial parts of *S. alpina* and *S. altissima* grown in a botanical garden in Poland. It also evaluates the inhibitory activity of the AGE formation of compounds identified as major bioactive metabolites from the analyzed extracts (baicalin, wogonoside, luteolin, luteolin-7-glucoside and verbascoside). They were identified in the *Scutellaria* samples by comparing their retention times, UV spectra and MS data with those of the standards. LC-MS/MS (liquid chromatography-mass spectrometry) data of the compounds has been published previously [14,15] (Tables S1 and S2, Supplementary Materials).

## 2. Results

In order to fully characterize the antioxidant properties of *Scutellaria* plants, three *in vitro* tests based on different mechanisms were used in the study (Table 1).

The FRAP assay measures the ability to reduce iron ions (Fe^3+^ to Fe^2+^). The strongest reducing ability was observed for the root extract of *S. alpina* (717.6 µM Fe(II)/g dry extract), which was significantly higher than shoot extract (669.6 µM Fe(II)/g dry extract) and almost twice as high as in extracts from *S. altissima* (369.4 and 391.7 µM Fe(II)/g dry extract), for roots and shoots, respectively).

In the DPPH anti-free radical assay, the extract from *S. alpina* roots again was the most efficient, with the lowest EC_50_ of 69 µg/mL. The values were found to be only a little higher in the shoots of this species (71.3 µg/mL), and the difference was not statistically significant. Both *S. altissima* extracts had significantly lower abilities to scavenge free radicals (EC_50_ = 82.9 and 102.7 µg/mL, for roots and shoots, respectively).

The inhibition of linoleic acid peroxidation (LPO) by *Scutellaria* plant extracts was assayed by the TBARS test. Similar to the DPPH and FRAP assays, the best inhibition ability was observed for the *S. alpina* root extract. The extract inhibited linoleic acid peroxidation by more than 60%. The shoots of *S. altissima* showed the weakest activity, with an inhibitory effect of 30%.

**Figure 1:**
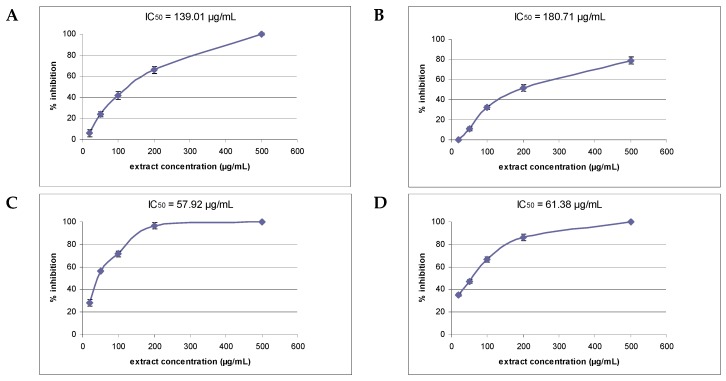
The inhibitory effect on advanced glycation end-products (AGE) formation of *Scutellaria altissima* shoot (**A**) and root (**B**) extracts and *S. alpina* shoot (**C**) and root (**D**) extracts at different concentrations. The results are mean values ± SE. IC_50_, the concentration (µg/mL) required to reduce fluorescent AGE formation by 50% (*n* = 4–5).

The ability of *Scutellaria altissima* and *S. alpina* extracts to inhibit AGE formation was evaluated using the antiglycation assay, in which bovine serum albumin served as the model protein and glucose and fructose as the glycating agents. The formation of AGEs was assessed by monitoring the production of fluorescent products at excitation and emission maxima of 335 nm and 385 nm, respectively [1]. Higher wavelength settings (370/440 nm) are unsuitable for *Scutellaria* extracts, due to the strong interference from autofluorescence. All tested extracts inhibited AGE formation in a dose-dependent manner. In general, *S. alpina* extracts showed a higher inhibitory effect than the extracts of *S. altissima*. Both *S. alpina* extracts presented more than 70% inhibition of AGE formation at a concentration of 100 µg/mL. These extracts exhibited IC_50_ values of about 60 µg/mL (57.9 µg/mL for shoots and 61.4 µg/mL for roots). *S. altissima* extracts showed much lower activity (IC_50_ 140–180 µg/mL) (Figure 1).

**Figure 2:**
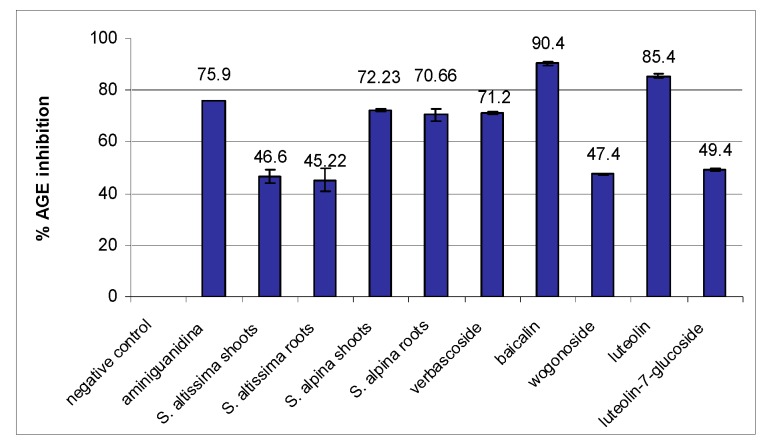
Antiglycation activity (expressed as % inhibition of AGE formation) of *S. altissima* (shoot, root) extracts, *S. alpina* (shoot, root) extracts and the main compounds present in the analyzed extracts (verbascoside, baicalin, wogonoside, luteolin, luteolin-7-glucoside). BSA after glycation (100% AGE) was used as the negative and aminoguanidine as the positive control. All extracts and compounds were used at a concentration of 100 µg/mL. The results are the mean values ± SE.

Having identified the main metabolites present in the analyzed extracts, *i.e*., four flavonoids (baicalin, wogonoside, luteolin, luteolin-7-*O*-β-d-glucoside) and one phenylethanoid (verbascoside), the next stage was to evaluate their antiglycation capacity. Baicalin exhibited the highest antiglycation activity, followed by luteolin. The samples inhibited 90.4 and 85% of AGEs at a concentration of 100 µg/mL (Figure 2). Aminoguanidine, used as a positive control, gave only 75% inhibition, similar to the phenylethanoid verbascoside (71%). Two other flavone glycosides (wogonoside and luteolin-7 glucoside) exhibited the lowest activity (about 50%).

A high correlation was observed between extract antiglycation activity and total flavonoid content (mg quercetin equivalents/g extract dry wt) (r = 0.99) (Table 2). Lower, but also significant, was the relationship between the inhibitory effect of AGE formation and verbascoside level (r = 0.73). Moreover, the antiglycation effect was strongly correlated with the antioxidant potential of the analyzed plant extracts, as demonstrated by the reducing (FRAP) and inhibition of lipid peroxidation (LPO) assays (r = 0.99 and 0.93, respectively). The antiradical test correlated slightly less with fluorescent AGE formation (r = 0.88).

## 3. Discussion

The results of the FRAP, DPPH and LPO assays revealed that all tested *Scutellaria* extracts have potent antioxidant activity. *S. alpina* extracts demonstrated similar EC_50_ values as *Zingiber officinale* (75 µg/mL), while *S. altissima* was comparable to *Myristica fragrans* (100 µg/mL) [16] and *Camelia sinensis* (100 µg/mL) [17].

The antioxidant properties of *Scutellaria* plants can be explained by the presence of different flavones and the phenylethanoid glycoside verbascoside, also known as acteoside. Both drugs from *S. alpina* are rich in flavonoids: they were found to contain about twice the amount as *S. altissima* (Table 1). A quantitative analysis of verbascoside present in extracts from the roots and shoots of *S. altissima* and *S. alpina* is given elsewhere [13]. This phenylethanoid glycoside was found in extracts from both analyzed *Scutellaria* species and was found in greater amounts in the roots than the shoots of both plants. Additionally, *S. alpina* accumulated higher verbascoside levels in both organs.

Numerous studies evaluating the antioxidant capacity of flavonoids have been published. The compounds have the ability to quench free radicals through the donation of electrons and hydrogen atoms, chelate transition metals or inhibit lipid peroxidation [18]. Verbascoside has also been shown to have antioxidant activity. It has been reported to display radical scavenging activity against diphenylpicrylhydrazyl (DPPH) and hydroxyl and superoxide anions [19].

In this study, BSA was chosen as the model protein for AGE formation. The intensity of fluorescence increased during the process of glycation, while the tested extracts and compounds suppressed this. The inhibitory effects of extracts from *S. alpina* and *S. altissima* plants were measured first. A representative antiglycation compound, aminoguanidine (AG), was used as a positive control. AG is known to prevent AGE formation by trapping intermediates at the initial glycation stages. However, its clinical use is limited by the presence of some side effects, such as drug resistance and hepatotoxicity [20]. All analyzed extracts exhibited good antiglycation ability, though *S. alpina* extracts showed higher potential (IC_50_ about 60 µg/mL) than aminoguanidine (63 µg/mL). The analyzed extracts displayed higher antiglycation activity than strawberry, blueberry or lemon fruit (IC_50_ between 290–460 µg/mL), although not as much as the peel of *Punica granatum* (5 µg/mL) or *Garcinia mangostana* (40 µg/mL) [21].

Glycation and AGE formation are associated with increased free radical production. Glycation is a major source of the reactive carbonyl and oxygen species generated by oxidative and non-oxidative pathways [22]. Inhibitors of AGE products may act not only as quenchers of dicarbonyl intermediates, but also as antioxidants or metal ion chelators. Therefore, compounds with antioxidant activity could also inhibit the formation of AGE. Nakagawa *et al.* [23] noted that green tea demonstrates strong antiglycation activity in addition to its known antioxidant potential. However, Chen *et al.* [24] describe plant extracts that possess strong antiglycation, but low antioxidant activity (*Astragalus membranaceus*), or strong antioxidant, but low antiglycation potential (*Periploca sepium*). Our present findings indicate a strong relationship between antioxidant properties and antiglycation activity (r = 0.89–0.99, depending on the antioxidant test). Inhibition of AGE formation correlated more with FRAP and LPO than with DPPH scavenging, which likely reflects the greater involvement of the reduction and peroxidation prevention than direct radical scavenging in glycation reactions. In some berry species, a higher correlation was observed between the inhibition of AGE formation and oxygen radical absorbance capacity (ORAC) than with the DPPH assay [25].

The antioxidant properties of many plant extracts are generally attributed to their phenolic content [26,27]. Several independent studies have demonstrated that antiglycation properties significantly correlate with the concentration of these compounds [28]. For example, Dearlove *et al.* [29] observed a strong relationship between antiglycation activity and total phenolic content in twenty-four herbs and spices. However, Ramkissoon *et al.* [30] proposed that the antiglycation properties of plant extracts cannot always be attributed to their phenolic content or antioxidant potential. Firstly, other compounds present in the extracts have been reported to interfere in fluorescence measurements [1]. Such interference was also seen in our analysis, when the intensity of fluorescence was measured at an extract excitation wavelength of 370 nm and emission wavelength of 440 nm. Secondly, the low correlation between antiglycation potential and polyphenol level may be associated with several possible mechanisms of activity, not only those associated with the antioxidant model, as confirmed by tests on quercetin and ascorbic acid. While quercetin exhibited both high antioxidant and antiglycation activities, vitamin C has no antiglycation ability, despite being a strong antioxidant [25].

All pure compounds tested in our study inhibited AGE formation, but their activity was significantly different. Baicalin and luteolin showed the greatest inhibitory effects. These two compounds were more effective glycation inhibitors than the reference drug aminoguanidine. It is known that the activity of flavonoids is strongly related to their structure. Studies on individual compounds reported by Matsuda *et al.* [31] provide structural determinants of both antiglycation and antioxidant activity. In particular, flavonoids with hydroxyl groups at the 3-′, 4′-, 5- and 7-positions showed a significant inhibitory activity against AGE *in vitro*. The activities of flavones were stronger than those of flavonols, flavanones or isoflavones. Furthermore, methylation or glucosylation of the 4′-hydroxyl group increased, but blocking of the 7-hydroxyl group reduced the antiglycation activity. The authors report a correlation between AGE inhibitory and scavenging activity. However, several conflicting observations were noted regarding antioxidant and antiglycation ability. For example, methylation of the 3-hydroxyl group enhanced the inhibitory effect concerning AGE formation, but reduced antiradical potential, as confirmed by the weaker activity demonstrated by 7-*O*-glucoside than aglycon luteolin observed in our study. Conversely, baicalin, which is also a 7-*O*-glycosylated (glucuronide) flavone with fewer hydroxyl groups, was stronger than luteolin. This is consistent with Matsuda *et al.* [31], who report that the baicalin aglycon, baicalein showed stronger inhibition than luteolin (79% and 64%, respectively, at a concentration of 200 µM, equivalent of roughly 54 and 57 μg/mL). The pivotal role of the hydroxyl group number can be also questioned by the comparable efficiency of luteolin-7-*O*-glucoside and wogonoside, even though the latter compound has only one free OH group. Therefore, other structural features contribute to the final antiglycation activity besides the number of hydroxyl groups. A more thorough study of structure-activity relationships, including their quantitative and mechanistic aspects, is necessary to explain the conflicting experimental observations.

Besides the flavonoids, verbascoside also has an influence on the antiglycation activity of *Scutellaria* extracts. Its ability to inhibit AGE formation was close to that of aminoguanidine. However, the correlation between the antiglycation potential of the studied extracts and the level of verbascoside was not as strong as for flavonoids. In *Brandisia hancei* (*Paulowniaceae*), verbascoside (acteoside) was the main antiglycative compound. It was found to be five-times stronger than quercetin (a flavonol) in terms of molar IC_50_ (5.11 μM *vs*. 28.41 μM), but only about 2.7-times greater in terms of mass concentration units [32]. Beside glycation and aldose reductase inhibition, verbascoside was able to decrease glucose level and increase glucose tolerance in mice [33]. However, no previous study could be found that compared the activity of verbascoside to other flavones thought to be stronger than flavonols.

## 4. Experimental Section

### 4.1. Plant Material

Plants of both species were grown for two years in the Medicinal Plant Garden of the Department of Pharmacognosy at the Medical University of Lodz. The plants were identified by Dr. I. Grzegorczyk-Karolak, and the voucher specimens were deposited in the Department of Biology and Pharmaceutical Botany, Medical University of Lodz. The *S. altissima* seeds were provided by the Garden of Medicinal Plants in Wroclaw (Wroclaw, Poland), and those of *S. alpina* were provided by the Botanical Garden of Institute of Ecology and Botany in Vácrátót (Vácrátót, Hungary). The experiments used the roots and aerial parts of the plants harvested during flowering. The plant material was cut and freeze dried.

### 4.2. Preparation of Extracts

The lyophilized plant material (1 g) was pre-extracted by maceration in chloroform overnight at room temperature. After filtration, the plant material was extracted three times with 30 mL methanol:water (7:3, *v*/*v*) for 15 min in an ultrasonic bath. The extracts were combined and evaporated under reduced pressure.

### 4.3. Chemicals

Standard compounds were used in the study, such as baicalin provided by Sigma-Aldrich (Darmstadt, Germany), wogonoside by ChemFace, luteolin and luteolin-7-*O*-glucoside (cynaroside) by Roth and verbascoside by Phytoplan.

### 4.4. Total Flavonoid Content

The colorimetric evaluation of flavonoid content was performed according to Lamaison and Carnat [34]. The absorbance of the reaction mixture was measured at 415 nm (Beijing Rayleigh Corp., Beijing, China). Quantification was performed using a standard quercetin (Sigma-Aldrich) calibration curve. The results were expressed as quercetin equivalents (mg per gram of dry extract).

### 4.5. DPPH Radical Scavenging Assay

The radical scavenging activity of plant extracts against the 1,1-diphenyl-2-picrylhydrazyl free radical (DPPH) (Sigma-Aldrich) was determined spectrophotometrically as described by Weremczuk-Jeżyna *et al.* [35]. The results were expressed as EC_50_ (µg/mL), calculated as the concentration of sample with 50% of maximum scavenging activity.

### 4.6. FRAP Assay

The FRAP assay was determined according to Pulido *et al.* [36]. The antioxidant activity was determined against a standard of known FRAP value, ferrous sulfate, calculated from a calibration curve with concentrations from 0–2000 µM. The antioxidant activity was expressed in µM Fe(II)/g of dry extract.

### 4.7. Linoleic Acid Peroxidation Inhibition

Linoleic acid peroxidation (LPO) inhibition was determined by the TBARS test according to Choi *et al.* [37]. The ability of a sample to inhibit the oxidation of linoleic acid is measured by spectrophotometry at 532 nm. The percentage of linoleic acid peroxidation inhibition was calculated using the following equation:

% inhibition = (Abs control − Abs sample − Abs extract) × 100/(Abs control – Abs extract)
(1)

where Abs control is the absorbance of reaction mixture containing methanol instead of an extract.

### 4.8. Antiglycation Assay

The glycation of BSA was performed according to Bhatwadekar and Ghole [38] with some modifications. Briefly, BSA (1 mg/mL) was incubated with 0.25 M fructose and 0.25 M glucose in 0.1 M phosphate-buffered saline (PBS), pH 7.4, in darkness at 50 °C for four days. Before incubation, the solution of plant extracts or pure compounds (verbascoside, baicalin, wogonoside, luteolin, luteolin-7-glucoside or a reference glycation inhibitor, aminoguanidine) dissolved in 50% DMSO was added to the mixtures. The formation of glycated BSA was determined using fluorescent intensity at an excitation wavelength of 335 nm and emission wavelength of 385 nm. The percentage of AGEs inhibition was calculated using the following equation:

% AGE inhibition = (F control − F control blank) × 100/(F extract – F extract blank)
(2)

where (F control – F control blank) is the difference between the fluorescent intensity of BSA incubated with or without glucose and fructose and (F extract – F extract blank) is the difference between the fluorescent intensity of BSA and sugars incubated with or without plant extracts.

### 4.9. Statistical Analysis

All results are presented as the average ± standard error (SE). The results were analyzed using the Kruskal–Wallis test. The level of significance was set at 5%. STATISTICA 10.0 (STATSoft, Krakow, Poland) software was used for calculations. Each series of antioxidant and antiglycation assays and flavonoid measurements was repeated two or three times, and each sample was measured in triplicate. EC_50_, IC_50_, correlation coefficients between the antiglycation assay and antioxidant assays, verbascoside and total flavonoid content were calculated using MS-Excel software.

## 5. Conclusion

Our study shows for the first time that extracts of *Scutellaria alpina* and *S. altissima* possess antiglycation activity *in vitro. S. alpina* root extracts had the highest inhibitory effect on AGE formation. Furthermore, the results from the FRAP, DPPH and LPO assays indicated the high antioxidant activity of *S. altissima* and *S. alpina* extracts. The antioxidant properties of these two *Scutellaria* species and flavonoids contained in the extracts are, at least in part, involved in the mechanisms of albumin glycation inhibition. Two flavones from *Scutellaria*, baicalin and luteolin, were highly effective in preventing AGE formation.

In conclusion, among other therapeutic applications, *Scutellaria* plants should be considered as sources of active compounds against glycation-associated complications in diabetes and some types of cancer, as well as offering anti-aging properties.

## Figures and Tables

**Table 1:** Total phenolic compounds and antioxidant activities of *S. altissima* and *S. alpina* extracts determined by reducing power (FRAP), the DPPH scavenging assay and inhibition of linoleic acid peroxidation (TBARS detection).

Plant Material	Total Flavonoid Content *	FRAP µM Fe(II)/g Dry Extract	DPPH Assay EC_50_ (µg/mL) **	% Inhibition Lipid Peroxidation
*S. altissima* shoots	14.01 ± 0.15 ^a^	391.66 ± 6.13 ^c^	102.68 ± 3.91 ^d^	29.76 ± 1.19 ^d^
*S. altissima* roots	13.83 ± 0.13 ^a^	368.37 ± 7.23 ^d^	82.94 ± 0.45 ^c^	36.90 ± 0.84 ^c^
*S. alpina* shoots	25.05 ± 0.43 ^b^	669.63 ± 11.07 ^b^	71.26 ± 0.34 ^b^	51.85 ± 3.75 ^b^
*S. alpina* roots	27.16 ± 0.87 ^b^	717.60 ± 1.35 ^a^	68. 98 ±0.30 ^a^	60.32 ± 1.61 ^a^

* Expressed as quercetin equivalents (mg per gram of dry extract). ** EC_50_, the concentration of the sample (μg/mL) showing 50% of maximal radical scavenging activity. The means with the same letter for the same assay do not differ significantly according to the Kruskal-Wallis test (*p* ≤ 0.05). The values are the means of six replicates ± SE.

**Table 2:** Correlation coefficients between the antiglycation activity of *S. altissima* and *S. alpina* extracts (expressed as % of inhibition of AGE formation) and antioxidant activity (in FRAP, DPPH and lipid peroxidation (LPO) assays), total content of flavonoid compounds and verbascoside content.

Correlation Coefficient (r)	Flavonoid Content	Verbascoside Content	FRAP	DPPH (EC_50_)	% Inhibition LPO
% inhibition AGE formation	0.99	0.73	0.99	−0.83	0.93
Linear regression	y = 2.05x + 17.57	y = 3.85x + 44.92	y = 0.08x + 15.71	y = −0.79x + 123.18	y = 0.98x + 14.72

## Supplementary Materials

The following are available online at www.mdpi.com/1420-3049/21/6/739/s1. Table S1: MS fragmentation of the investigated compounds (verbascoside, baicalin, wogonoside) by HPLC-ESI-MS/MS, Table S2: LC-MS/MS spectrum of luteolin and luteolin-7-glucoside.
