# Supplementary material for: Inhibition of Advanced Glycation End-Product Formation and Antioxidant Activity by Extracts and Polyphenols from Scutellaria alpina L. and S. altissima L
Source: Molecules. 2016 Jun 14;21(6):739. doi: 10.3390/molecules21060739 (PMC6273165; doi:10.3390/molecules21060739)
Supplement: Supplementary file 1 [file molecules-21-00739-s001.pdf]

Supplementary Materials: Inhibition of Advanced Glycation End-Product Formation and Antioxidant Activity by Extracts and Polyphenols from *Scutellaria alpina* L. and *S. altissima* L.

Izabela Grzegorczyk-Karolak, Krzysztof Gołąb, Jakub Gburek, Halina Wysokińska and Adam Matkowski

Liquid Chromatography-mass Spectrometry (LC-MS/MS)

LC-MS/MS was carried out using API LC/MS/MS system (Applera, Norwalk, Conn., USA) with electrospray ionization (ESI) source equipped with Dionex (Dreieich, Germany) HPLC system. Separation of the compounds was achieved on Hypersil Gold RP column (C18, 2.1 mm × 150 mm, 5 μm) protected by Hypersil Gold C18 guard column (2.1 mm × 10 mm, 5 μm) at 27 °C. Detection was performed in both positive and negative ion modes with the conditions set as follows: drying gas (N_2) 11.0 L/min, temperature 350 °C, nitrogen nebulizer pressure 40 psi, capillary voltage 4.0 kV, a detector gain of 1600 V, fragmentation voltage 100 V and full scan range from 100 to 900 *m*/*z*.

Table S1. MS fragmentation of the investigated compounds (verbascoside, baicalin, wogonoside) by HPLC-ESI-MS/MS.

Compound	MS Ions (*m*/*z*)	
	Positive Ion Mode	Negative Ion Mode
verbascoside	625.2 [M + H]$^+$	623.2 [M − H]$^-$; 669.6 [M + HCOO]$^-$; 461.2 [M − H − CaA]$^-$
baicalin	447.3 [M + H]$^+$; 271.1 [M + H − GlA]$^+$	445.2 [M − H]$^-$; 269.1 [M − H − GlA]$^-$
wogonoside	461.4 [M + H]$^+$; 285.1 [M + H − GlA]$^+$	459.3 [M − H]$^-$; 283 [M − H − GlA]$^-$

Table S2. LC-MS/MS spectrum of luteolin and luteolin-7-glucoside.

Compound	MS Ions (*m*/*z*)
luteolin	285.2 [M − H]$^-$; 241.2 [M − H − CO_2]$^-$; 175.1 [M − H − C_3O_2-C_2H_2O]$^-$; 199.2 [M − H − C_2H_2O − CO_2]$^-$; 243.2 [M − H − C_2H_2O]$^-$; 217.2 [M − H − C_3O_2]$^-$; 174.7 [M − H − catechol]$^-$; 257.2 [M − H − CO]$^-$; 213.2 [M − H − CO_2 − CO]$^-$
luteolin-7-*O*-glucoside	447.1 [M − H]$^-$; 285.1 [M − H − Glu]$^-$; 174.7 [M − H − Glu − catechol]$^-$; 241.1 [M − H − Glu − CO_2]$^-$; 175.1 [M − H − Glu − C_3O_2 − C_2H_2O]$^-$; 199.2 [M − H − Glu − C_2H_2O − CO_2]$^-$; 243.2 [M − H − Glu − C_2H_2O]$^-$; 217.2 [M − H − Glu − C_3O_2]$^-$
